# Critical Features of Fragment Libraries for Protein Structure Prediction

**DOI:** 10.1371/journal.pone.0170131

**Published:** 2017-01-13

**Authors:** Raphael Trevizani, Fábio Lima Custódio, Karina Baptista dos Santos, Laurent Emmanuel Dardenne

**Affiliations:** 1 Fiocruz, Fundação Oswaldo Cruz, Fortaleza, Ceará, Brazil; 2 Grupo de Modelagem Molecular de Sistemas Biológicos, Laboratório Nacional de Computação Científica, Petrópolis, Rio de Janeiro, Brazil; University of Michigan, UNITED STATES

## Abstract

The use of fragment libraries is a popular approach among protein structure prediction methods and has proven to substantially improve the quality of predicted structures. However, some vital aspects of a fragment library that influence the accuracy of modeling a native structure remain to be determined. This study investigates some of these features. Particularly, we analyze the effect of using secondary structure prediction guiding fragments selection, different fragments sizes and the effect of structural clustering of fragments within libraries. To have a clearer view of how these factors affect protein structure prediction, we isolated the process of model building by fragment assembly from some common limitations associated with prediction methods, e.g., imprecise energy functions and optimization algorithms, by employing an exact structure-based objective function under a greedy algorithm. Our results indicate that shorter fragments reproduce the native structure more accurately than the longer. Libraries composed of multiple fragment lengths generate even better structures, where longer fragments show to be more useful at the beginning of the simulations. The use of many different fragment sizes shows little improvement when compared to predictions carried out with libraries that comprise only three different fragment sizes. Models obtained from libraries built using only sequence similarity are, on average, better than those built with a secondary structure prediction bias. However, we found that the use of secondary structure prediction allows greater reduction of the search space, which is invaluable for prediction methods. The results of this study can be critical guidelines for the use of fragment libraries in protein structure prediction.

## Introduction

It is estimated that less than 30% of human protein structures have been empirically determined, and the amount for other species is significantly lower [1]. This is primarily due to the difficulties and costs associated with experimental techniques, such as NMR and X-ray diffraction. Meanwhile, genomes are sequenced at relatively low cost, increasing the gap between known sequences and known structures motivating the development of computational tools for protein structure prediction (PSP). Among such tools, comparative modeling yields the best results when the target sequence has a high degree of sequence similarity with proteins available in structural databases. When such homologous structures are not available, an *ab initio* approach is required.

*Ab initio* methods consist in predicting the native structure of a protein from its amino acid sequence alone. It is a formidable computational challenge usually associated with hundreds of degrees of freedom and complex energy functions in an attempt to model the intricate interplay of forces stabilizing the protein structure. The computational cost for sampling and evaluating a very complex energy landscape to identify the global minimum is too high even for small proteins.

The description of the secondary structures [2, 3] and regular turns [4, 5] helped to reduce the complexity of the PSP problem. Later, the pioneering work of Jones and Thirup [6] lead to the discovery that a single portion of a protein could be modeled using fragments from other proteins, regardless of any apparent evolutionary correlations. The fragments structures varied from secondary structures and simple coils to parts of two secondary structures joined by a loop [7–9].

Currently, many successful PSP methods such as ITASSER [10–12], ROSETTA [13] and QUARK [14] use fragment libraries to boost the accuracy and reduce the number of degrees of freedom during the conformational search. The combination of *ab initio* prediction and fragment libraries is only possible because fragments are not restricted to closely related proteins [15]. Han and Baker [16] suggested that a single structure can be associated with more than one sequence, and found a pattern which correlates sequences of fragments to local motifs. The authors showed that motifs were coded by sequences of amino acids sharing common properties such as charge, the presence of aromatic residues and, mainly, hydrophobicity.

Although fragments are stable throughout different proteins and protein families, as they are part of a protein rather than an isolated molecule, they are subject to different forces when the chemical environment is changed. Therefore, the same sequence may code for different structures. This multiplicity of structural states for a single sequence and the fact that the same structure can be found in various non-homologous proteins [17, 18] are the key to PSP methods using fragments. Since a fragment represents a likely local minimum of the potential energy function, its use allows the protein to jump from one conformation to another, thus exploring the energy landscape more effectively than a gradient-based optimization or a molecular dynamics simulation [13].

Fragment libraries are also considered valuable tools for loop predictions. Loop structure prediction is regarded as a difficult task since loops are subject to more structural diversity, as they are the main components of protein surfaces and are frequent in binding sites. Some authors report promising results in loop prediction using smaller fragments because, although not optimally representing an entire loop, they helped reduce the number of conformations to be evaluated [19, 20].

Levitt [21], Kolodny and collaborators [22] successfully rebuilt a protein from a collection of small fragments. Holmes and Tsai [23] evaluated the ability of different fragment libraries to model the native structure concerning cost and accuracy of building models in Cartesian space versus torsion space, and the effects of the first and last angles of the inserted fragment on the overall structure. The authors also showed a significant increase in model quality when fragment bond angles and bond distances were added to the models, as opposed to solely using dihedrals. There is also evidence that no fragment size is ideal, and it has been suggested that one needs libraries with different fragment sizes.

Xu and Zhang [24] investigated several criteria for optimal fragment libraries, such as predictions of residue–residue contacts derived from distance profiles, torsion angle prediction, optimal number of fragments per position, secondary structure prediction and sequence similarity. Among these, the most relevant were proven to be secondary structure prediction and sequence similarity. The author concluded the fragment of 10 residues lead to the best results and that the library should have least 100 fragments per position.

In search of the optimal fragment length, Handl and collaborators report that larger fragments are surprisingly useful, and it has been proposed that fragments of smaller sizes should be derived from them [25]. The work also supports the importance of smaller fragments in refining the structure, in particular, *β*-sheets, and recommends further investigation on which attributes result in a better fragment library. From a set of 1261 non-redundant proteins, Baeten and collaborators [19] derived the Brix database composed of 4-14 residues long fragments. Brix contains 1000-2000 conformations for each fragment length. Using only the backbone Root Means Square Deviation (RMSD) as a scoring function and without any fragment selection criterion, i.e., using the whole fragment database, close to native structures were built. They showed that loop regions could be better reconstructed using smaller fragments, although regular secondary structures were best approximated by larger fragments. Later, it was reported that the variability of loops connecting helices is greater than those connecting *β*-strands [26].

To further integrate structural constraints, it has been proposed that better fragment selection could be achieved if protein class information were taken into account [27]. The authors integrated class annotations predicted from sequence alone to fragment libraries and used the Rosetta *ab initio* protocol to model native structures, reporting up to 7% improvement in model quality.

To sample as many feasible conformations as possible, a library must contain as many structurally diverse fragments as possible, avoid redundancies and, if possible, include fragments that, at least locally, strongly correlate with the target sequence. Usually, the score from a substitution matrix (e.g., BLOSUM) is used to select which fragments will be contained in a library, and several PSP methods use secondary structure predictors as an additional criterion to select fragments. One of the most commonly used secondary structure predictor is PSIPRED [8], which has been especially successful since CASP4 when it reached an 80.6% success rate for 40 protein domains [28]. However, to our knowledge, it has never been fully evaluated how or to which extent secondary structure prediction increases the predictive potential of fragment libraries.

Fragment libraries have been shown to work well in practice by greatly improving the quality of the predicted structures, but considering how current PSP methods rely on fragment libraries, it is important to obtain guidelines for their use and construction. Despite being commonly used, some essential aspects of fragment libraries that influence the correct modeling of native structures remain to be determined. This study investigates some crucial features of a fragment library in the reconstruction of the native structure, isolated from the limitations of *ab initio* methods, such as an imprecise energy function and optimization algorithms. Concretely, we addressed the extent to which the following features affect the assembly of tertiary structures: (1) secondary structure prediction guiding fragments selection, (2) different fragments sizes and (3) the structural clustering of fragments within libraries. All the simulations were performed with fragments excised from structures that are non-homologous to the targets, therefore emulating a free-modeling prediction.

## Results and Discussion

For each protein in the test set, 30 models were built from libraries comprising fragments of 3 to 20 residues long, selected by their sequence similarity with the target protein (given by the score of BLOSUM62) combined with their secondary structure prediction (given by PSIPRED confidence). These libraries were built using the Profrager server [29] (https://www.lncc.br/sinapad/Profrager).

We adopted the RMSD of the main chain atoms (N, C*α*, C) against the structure deposited in the PDB as the measure to evaluate the accuracy of the models. For small proteins, a model is often regarded as good if the RMSD is below 3 Å, acceptable when below 5 Å and uninformative when above 5 Å [30].

### Single-size fragment libraries

**Fig 1:**
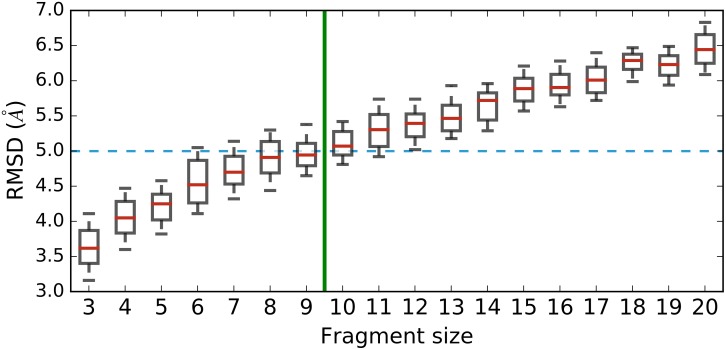
RMSDs of the best models of single-length, PSI libraries. The blue line marks the RMSD cutoff for an acceptable model. Libraries on the left of the green line generated at least 50% models below the RMSD cut-off. Outliers not shown.

The distribution of RMSDs of the models created by each individual library shows smaller fragments have the potential to generate more accurate results (Fig 1).

Our data shows that none of the models done with 12-mers or larger, and few of the models done with 10-mers to 12-mers meet the RMSD criterion for acceptable models. Even though longer fragments may bring more empirical information into the model, thus further constraining the search space and speeding the convergence towards the native state, the models built purely from longer fragments were of lesser quality than those built from smaller fragments.

There are two possible reasons for the inferior quality of the models assembled with longer fragments. The first is that they might be insufficiently well-represented in databases since the structural diversity grows with the number of residues [19]. Fragments in regions of *α*-helices have more quality when compared to those of regions of *β*-sheets, which probably makes longer fragments unsuitable to correctly model *β*-sheets [31]. Ergo, longer fragments are of lesser quality because known proteins lack a suitable structure for the target sequence.

The second reason is that the insertion of a longer fragment when the conformation is close to native may bring changes that, though locally favorable, could be too drastic for the rest of the structure. Taken together, the lack of diversity and the stronger perturbation may account for the less accurate models obtained using longer fragments.

Fragments with 9 residues or smaller generated satisfactory models for at least half of the proteins studied (Fig 1). Fragments larger than 9 have been supported elsewhere, but the tests always involved a combination of large fragments of many different sizes, as well as smaller fragments ([14, 19, 24]). Our data reveals the use of fragments that large may have been overrated in the literature as they show limited predictive capacity when used isolatedly.

Given that fragments longer than 9-mers may be disregarded in fragment libraries, there is also the question of whether it is possible to remove other sizes without negatively impacting the overall quality of the final models. There is a significant difference between the results of 3-mers and 9-mers (*p* < 0.01), which means that larger fragments cannot reproduce the native structure with the same accuracy as smaller fragments. Nonetheless, 9-mers have been shown to improve model accuracy when combined with fragments of other sizes. Rosetta, for example, is a well-established method for protein structure prediction that traditionally uses 9-mers and 3-mers in their ab initio predictions. However, there are no significant differences between the RMSDs of the models generated by 4-mers to 8-mers (*p* = 0.011), suggesting a certain degree of structural redundancy in these fragments. These results could prove valuable for simulations with mixed libraries, *ie*, libraries that combine several fragment sizes.

### Mixed libraries

A series of different works demonstrate the use of fragments of different sizes generates closer to native models [14, 32]. In this section, we focus on the analysis of the mixed-size libraries to obtain some insights about how to construct them, based on the results of the tests with the single-size libraries.

Since 3-mers and 9-mers are statistically apart from each other, we selected the 9-mer to represent the ″large fragment‶ and 3-mer to represent the ″short‶. Because 3-mers and 9-mers generate models that are significantly different, we found prudent to bridge the gap between them with a fragment of intermediate size. In view of the fact that fragments with 4 to 8 residues do not produce models that are significantly different, any size is equally fit to be considered ″medium‶. We selected a 6-mer as the medium-sized fragment.

**Fig 2:**
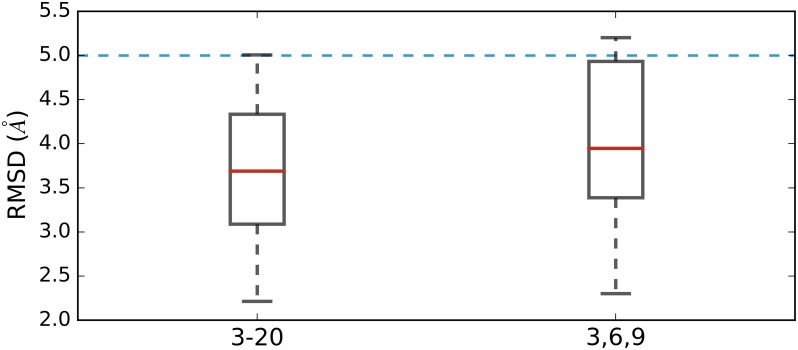
Comparison of best models. 3-20: RMSD distribution of the best models assembled by PSI fragments of 3 to 20 residues long. 3,6,9: RMSD distribution of the best models assembled by 3-mers, 6-mers and 9-mers of PSI libraries.

To verify if a mixed library exclusively composed of 3,6,9-mers is adequate to reproduce the native structure, we generated a new set of models and compared them with those produced by mixed libraries with 3-mers to 20-mers. The comparison of the RMSDs of the best models created with 3,6,9-mers shows no significant difference to those created with 3-20-mers (Fig 2, *p* = 0.14), which indicates there is no need to add different sizes and that three different fragment sizes are sufficiently diverse to satisfactorily rebuild the native structure.

**Fig 3:**
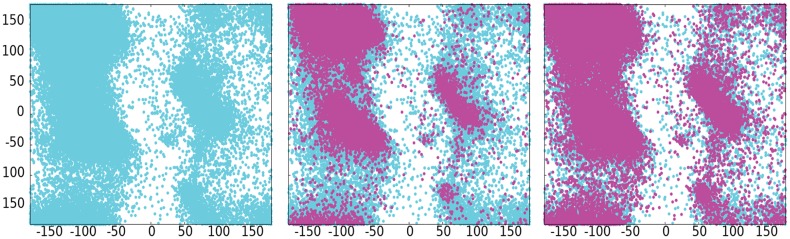
Ramachandran plot. Left: Ramachandran plot for 1E0L of PSI libraries 3-20-mers. Middle: Superposition of the Ramachandran plot for 3-20-mers (blue) and 3-mers (purple). Right: Superposition of the Ramachandran plot for 3-20-mers (blue) and 3,6,9-mers (purple). Similar plots are obtained for all the other protein fragment libraries.

This proves only a few fragment sizes are enough to model the native structure. All other sizes only seem to add structural redundancy without bringing any useful information that is worth a costly extension of the search space. When the Ramachandran plot of 3-mers to 20-mers and 3,6,9-mers are superposed, 3,6,9-mers cover most of the Ramachandran of libraries with sizes 3-20 (Fig 3).

In this case, we have shown 3,6,9-mers sufficiently cover the associated fragment conformational space, but this is by no means restricted to this specific combination of fragments. Perhaps other authors may find a different combination of sizes more suitable for a particular PSP methodology. It is also worth mentioning that with the increase of data deposited in the PDB, it is expected the barrier for greatest fragment size to surpass 9. For all evaluations in the following sections, we discarded all fragments other than 3,6,9-mers.

The fact that the majority of the best models are obtained with mixed libraries indicates that mixed libraries combine desirable properties of longer and shorter fragments [19, 20, 23, 33, 34]. Despite the disadvantages of 9-mers when compared to 3-mers and 6-mers, our findings suggest they have desirable, seemingly indispensable properties for better model accuracy when used in combination with different fragment sizes. Considering that it is theoretically possible to obtain a 9-mer by stringing three 3-mers, it seems counterintuitive that models built exclusively from 3-mers do not have the same quality of those built from mixed libraries. This is because simply adding 3-mers together does not necessarily lead to a naturally existing, clash-free 9-mer. Moreover, if there are 200 fragments per position and three positions, searching the correct 9-mer from a set of 3-mers requires 200^3^ evaluations instead of 200 evaluations.

In a previous work by Handl and collaborators [25] it was shown that longer fragments worked well for most proteins, which lead to the suggestion that small fragments such as 3-mers should be derived from the longer. However, extracting shorter fragments from the longer could greatly scale down structural diversity, thus undermining the usefulness of short fragments. Our simulations with the mixed libraries corroborate there is no ideal fragment size. Rather, the best approach seems to be the combination of different sizes, as it allows shorter fragments to compensate for shortcomings of the longer, and vice-versa.

**Fig 4:**
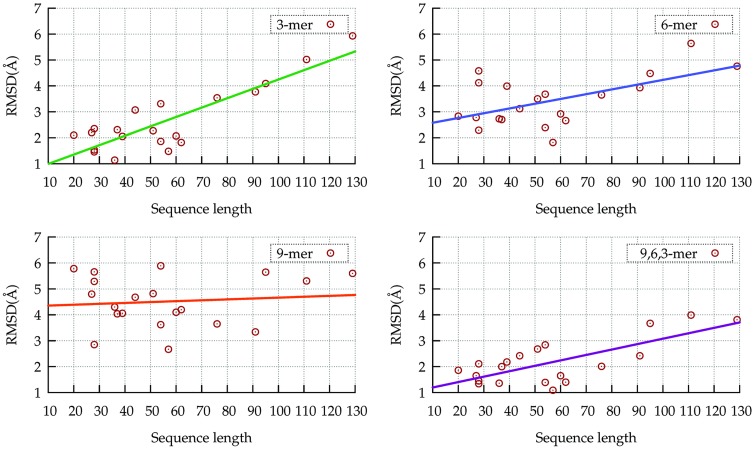
RMSDs of the best models. Comparison of the RMSD of the best models built only from 3-mers (upper left), only 6-mers (upper right), only 9-mers (bottom left) and 9,6,3-mers (bottom right).

The superiority of the mixed libraries is confirmed by the tests for which single-length libraries were incapable of generating proper protein models (Fig 4). Whereas models up to 50 residues built from 3-mers have approximately the same quality of those built from mixed libraries, the RMSDs for models built exclusively from 3-mers increase greatly with the target length (Fig 4). Such significant loss of quality is not observed for the mixed libraries.

**Fig 5:**
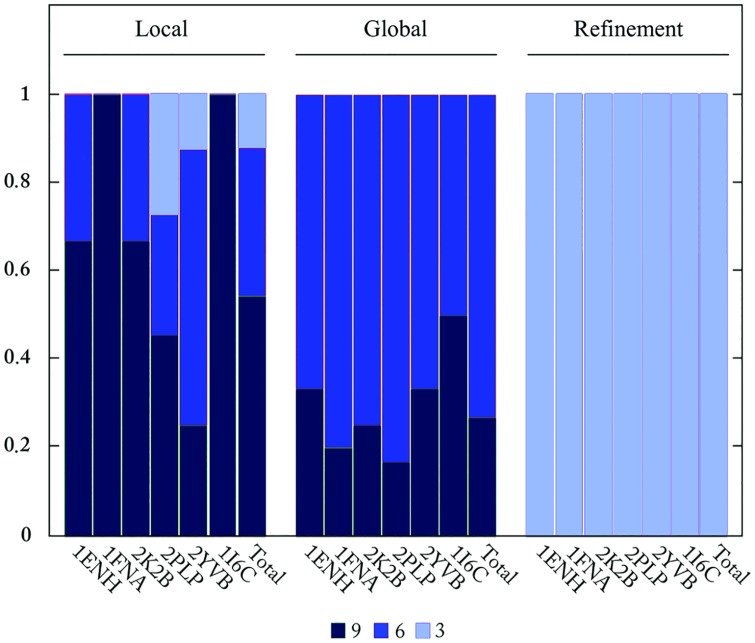
Relative frequency of fragment insertion. The bars show the relative amount of use of each fragment size (9-mers, 6-mers and 3-mers) in different stages of the algorithm when mixed libraries are used.

As mixed libraries contain fragments of all three sizes, it is important to describe *how* these fragments are used to built the best models. We observed that the best models constructed with mixed libraries were obtained by assembling 9-mers, followed by 6-mers and finally 3-mers (Fig 5, S1 Fig). In the course of the simulations with mixed libraries, 9-mers are especially inserted during the first stage of the optimization, because they bring more empirical information into the model, enabling the optimization algorithm to bias the structure towards the global minimum basin. However, 9-mers are expected to become less useful as the model approaches the native conformation possibly because they might cause structural changes that are inconvenient for the latter stages of the algorithm when more subtle changes are required.

As the simulation progresses, smaller fragments begin to play a major role, and in contrast, 9-mers start losing their importance. Because the global optimization is implemented to take into consideration the whole structure, the fragments used in this stage are somewhat likely to avoid radical conformational changes. Thus, smaller fragments become more suitable for PSP methods. As 6-mers are the most used fragments during global optimization, they apparently represent the best trade-off between the amount of information brought by larger fragments while still allowing for the minimal perturbation that is expected of smaller fragments.

As previously stated, the further into the simulation, the smaller was the fragment size, so it was expected that the final stage was characterized by the predominance of the smallest of all sizes, the 3-mer. In fact, the algorithm inserted 3-mers almost exclusively, indicating they are ideal to local structural refinement (Fig 5, S1 Fig).

### Use of secondary structure prediction

To verify how useful secondary structure prediction is for fragment libraries, we compared the models created by assembling fragments ranked according to sequence similarity alone (BLO) and fragments that combine sequence similarity and secondary structure prediction (PSI).

**Fig 6:**
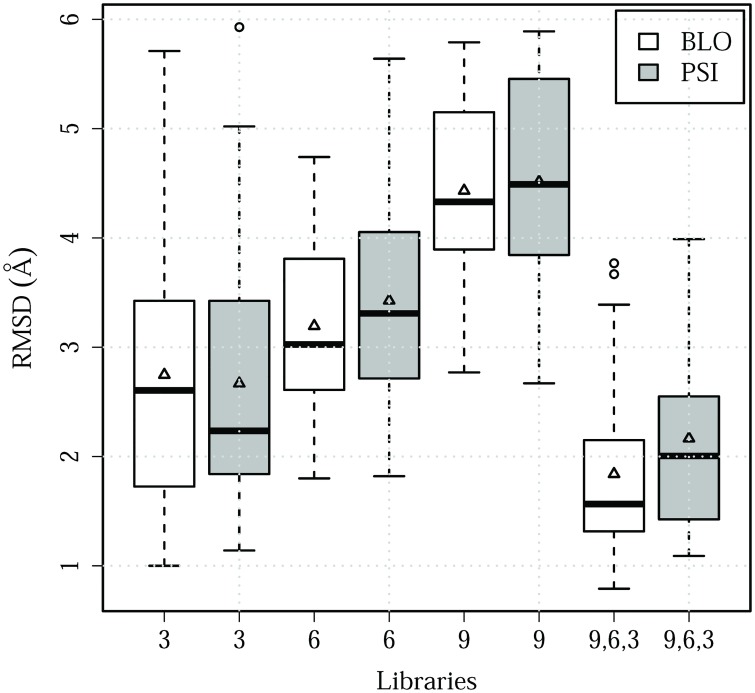
RMSDs of the best models. Models were built from a combination of different fragments length (9, 6 and 3-residues long) and scores (BLOSUM62, shown in white; PSIPRED+BLOSUM62, in gray).

**Fig 7:**
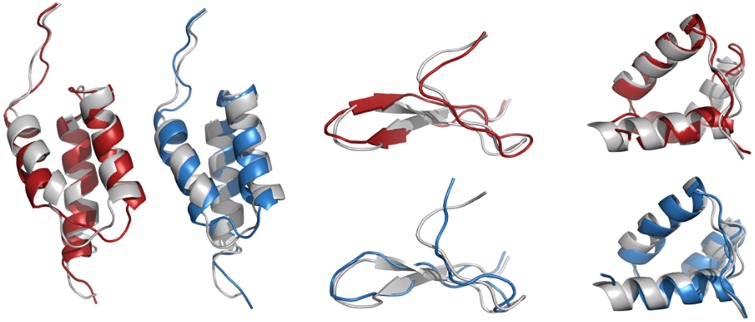
Best models obtained with the simulations with the mixed libraries. Models obtained with PSI libraries (blue) and BLO libraries (red) are superposed to the experimental structure (white). PDB(RMSD BLO, RMSD PSI): 1BDD (1.38,1.65); 1E0N (1.37,1.65), 1ENH (1.31,1.39).

**Fig 8:**
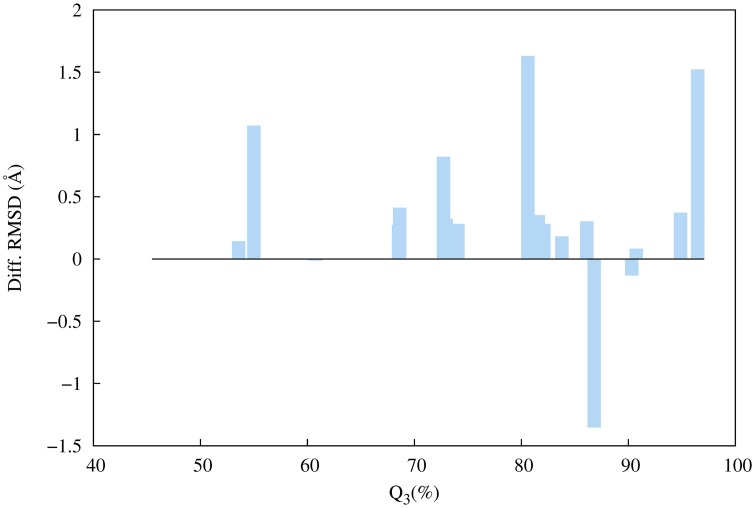
Effect of PSIPRED success rate (%) on the RMSD of the best models. If the difference is greater than zero (*Dif*_*RMSD*_ > 0) it means the use of PSIPRED is harmful to the prediction. Diff RMSD = Difference between the RMSD of PSI and BLO62 models.

Secondary structure prediction is widely used by nearly all the successful PSP methods as it has been found to unequivocally ameliorate model quality. Thence, it came as a surprise that the majority of our PSI libraries models are less accurate than the BLO models (Figs 6 and 7, S1 Table). It is important to notice that this is true regardless of the quality of PSIPRED prediction even when its success rate is close to 100% (Fig 8). Certainly, PSI models are expected to have lower quality when the PSIPRED predictions are inaccurate, as is the case with 1AMB, where only 14% of the PSIPRED prediction is correct, but PSIPRED had over 50% success ratio for all tested proteins.

The quality loss of the PSI models compared to BLO models despite the high rate of success in PSIPRED predictions strongly indicates the exclusion of essential fragments and raises questions over the ubiquitous use of secondary structure prediction in fragment selection.

**Fig 9:**
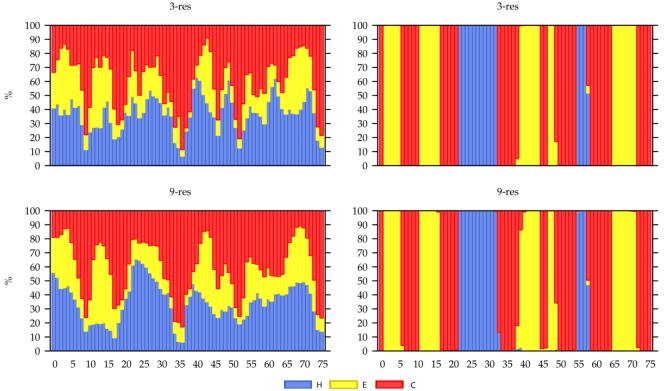
Secondary structure distribution profile for Profrager generated libraries with fragments containing 3 and 9 residues, target T0551. The horizontal axes represent each residue in the sequence. Vertical axes show secondary structure percentages among the fragments at each residue (position): H = helix, C = coil and E = extended. Left panel: BLO libraries built using only sequence similarity. Right panel: PSI libraries built using sequence similarity score and secondary structure prediction agreement score. These graphics are automatically generated by Profrager.

Are there any advantages in using secondary structure prediction in a PSP method? The search space of the problem of assembling a native structure from overlapping fragments consists of the many combinations of the dihedral angles found in the library. When fragment selection is biased by PSIPRED, a library with less structural diversity is created (Fig 9), so it spans a narrower search space. Despite the loss of desirable fragments, a more limited search space allows optimization algorithms to perform better.

The fragments were clustered based on their structural similarity to evaluate to which extent the use of secondary structure prediction allows the optimization algorithm to constrain the search space and how it influences protein modeling. Structural clustering consists in grouping the fragments according to a distance function (e.g.: RMSD, DME) when the value is within a cutoff. The expectation is to reduce the search space with a negligible loss in accuracy. The libraries were clustered by structural similarity, and new models were built using only the fragments with the greatest score from each cluster (referred to as *leader*).

**Fig 10:**
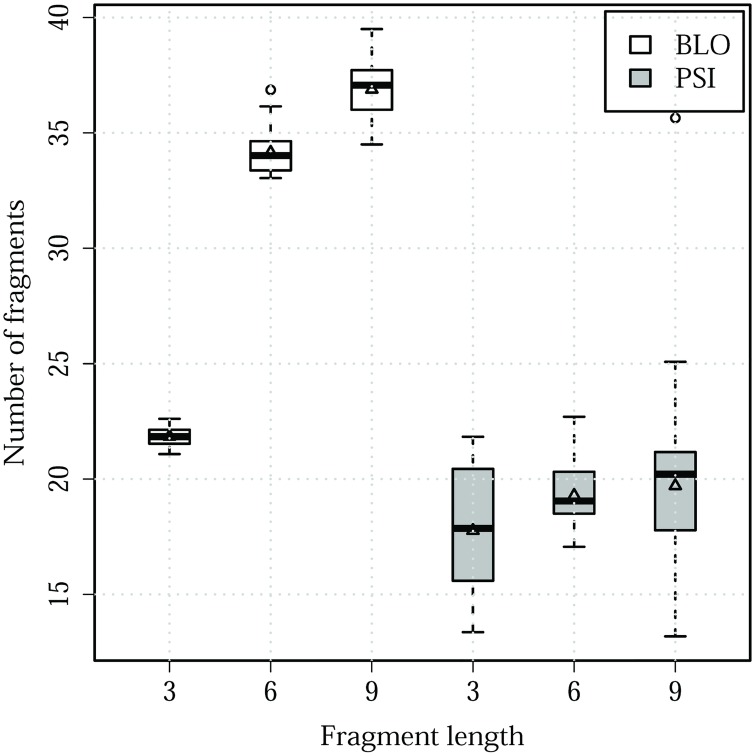
Number of clusters for each single-sized library of 3-mers, 6-mers, 9-mers.

Since PSI libraries are structurally less diverse, clustered PSI libraries resulted in approximately half the number of leaders of the BLO libraries (Fig 10).

There is a minor loss (at most 1.39 Å, Table 1) in accuracy when models are generated using only leaders, but such loss is compensated by the notable reduction of the search space from 200 fragments per residue to approximately 20 (Fig 10).

**Table 1:** RMSD difference between models from clustered and unclustered libraries.

PDB	N	BLO	PSI
1L2Y	20	0.23	0.06
1E0N	27	-0.22	0.38
1AMB	28	0.54	-0.93
1FSD	28	-0.01	-0.01
1PSV	28	0.15	0.15
1VII	36	0.21	-0.01
1E0L	37	-0.16	0.41
1I6C	39	0.44	0.41
2P81	44	0.37	-0.39
1BAL	51	0.55	0.75
1ENH	54	0.22	0.21
2PLP	54	0.12	0.74
1FYJ	57	0.20	0.75
1BDD	60	0.09	0.08
1KOY	62	1.39	0.37
2NR2	76	0.93	0.03
1FNA	91	0.40	1.39
1H5P	95	0.30	1.36
2K2B	111	0.55	1.31
2YVB	129	0.31	-0.25

The difference between RMSDs of the best models built from clustered and unclustered BLO and PSI libraries. Positive values indicate that the models from the clustered libraries have a larger RMSD, and negative values the opposite. N = number of residues. RMSD values are in relation to the backbone of the native structure.

While most state-of-the-art protein structure prediction software use one or more secondary structure prediction methods, our previous results of unclustered PSI and BLO (Fig 6) shows that the use of secondary structure prediction has no effect in selecting better fragments. However, clustered PSI libraries resulted in fewer clusters while maintaining similar accuracy when compared to BLO models. In a template-free prediction scenario, the use of PSIPRED allows the optimization algorithm to explore the conformational landscape more efficiently due to the resulting smaller number of clustered fragments.

By comparing the results of clustered and unclustered libraries, we suggest that the use of secondary structure prediction does not rank fragments in a way that selects closest-to-native local conformations. Instead, we suggest the use of secondary structure prediction is relevant to PSP methods because it effectively reduces the search space by sparing only the fragments with a similar secondary structure. The fact that BLO unclustered libraries are superior to PSI unclustered libraries indicates that occasionally the use of PSIPRED sacrifices potentially relevant fragments. However, regarding cost-effectiveness, using PSIPRED for fragment selection is a beneficial strategy due to the relatively smaller number of clusters obtained from PSI libraries.

### Validating libraries with Rosetta

As Profrager outputs files that are compatible with Rosetta, Rosetta was used to simulate a real *ab initio* scenario with BLO, PSI (9,3-mers, as used by Rosetta) and Robetta fragments. Robetta libraries were built using the Robetta server (http://robetta.bakerlab.org/fragmentsubmit.jsp).

Table 2 compares the GDT-TS values of the best models created using the three different fragment libraries.

**Table 2:** GDT-TS score (%) of the best models.

CASP9 ID	PDB	BLO	PSI	Robetta	Length
T0522	3nrd	28.85	41.54	42.31	134
T0523	3mqo	27.48	40.77	42.79	120
T0527	3mr0	24.61	29.33	32.09	142
T0530	3npp	33.43	44.77	58.43	115
T0531	2kjx	33.85	41.54	43.46	65
T0538	2l09	60.08	84.27	82.66	54
T0539	2l0b	22.25	21.70	20.60	81
T0540	3mx7	46.67	57.50	57.50	90
T0541	2l0d	28.95	40.13	36.40	106
T0544	2l3w	33.74	35.66	46.50	135
T0546	2l5q	27.29	32.39	29.93	134
T0548	3nnq	36.96	51.09	45.38	106
T0549	2kzv	40.49	50.00	55.16	84
T0551	3obh	28.13	28.52	31.25	74
T0552	2l3b	29.81	33.85	41.73	122
T0553	2ky4	28.69	40.10	40.60	141
T0555	2l06	28.23	27.42	34.19	148
T0557	2kyy	26.31	32.03	34.15	145
T0559	2l01	47.40	66.88	77.92	69
T0560	2l02	49.70	61.28	68.60	74
T0562	2kzx	32.44	37.98	39.89	123
T0564	2l0c	26.80	41.49	41.75	89
T0567	3n70	21.07	24.82	26.07	145
T0569	2kyw	37.93	38.51	38.79	79
T0572	2kxy	25.00	26.50	29.00	93
T0574	3nrf	26.49	32.18	41.83	126
T0579	2ky9	19.32	21.78	22.92	124
T0580	3nbm	35.89	47.28	47.52	105
T0581	3npd	40.77	38.96	43.69	136
T0586	3neu	28.91	36.52	33.48	125
T0590	2kzw	24.14	18.28	26.90	137
T0592	3nhv	19.70	19.89	17.99	144
T0594	3ni8	22.68	22.50	20.54	140
T0600	3nja	25.48	25.48	26.92	125
T0602	3nkz	41.40	47.04	50.27	123
T0605	3nmd	53.85	62.50	62.50	72
T0612	3o0l	33.41	36.45	40.89	129
T0614	3voq	24.35	23.71	24.57	135
T0616	3nrt	32.78	39.17	39.72	103
T0617	3nrv	27.38	34.13	37.50	148
T0619	3nrw	36.52	29.17	34.31	111
T0622	3nkl	40.21	60.83	57.71	138
T0624	3nrl	41.04	51.49	55.97	81
T0630	2kyt	26.40	31.20	37.40	132
T0634	3n53	25.00	34.48	42.46	140
T0637	2x3o	22.33	26.53	23.47	146
T0639	3nym	28.54	32.08	34.96	128
T0643	3nzl	51.43	58.21	63.21	83
Average		32.38	38.75	41.33	
GDT > 50		3	10	11	

**BLO: built using only sequence similarity (BLOSUM library)**. PSI: built using sequence similarity score and secondary structure prediction agreement (PSIPRED library). Robetta: libraries built using Robetta server.

**Fig 11:**
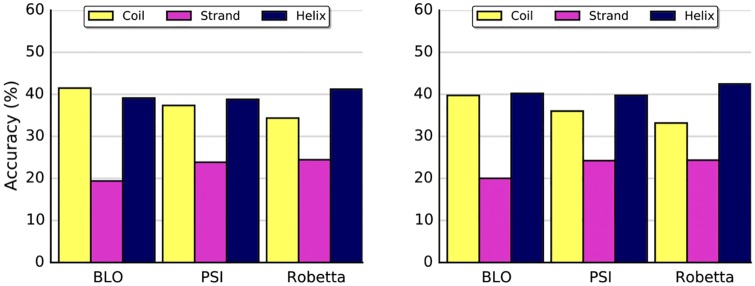
Ratio of correct secondary structures. Percentage of residues with the correct secondary structure in each fragment library of 3-mers (left) and 9-mers (right). For each residue, on the native structure, its secondary structure is compared to all corresponding fragments from the libraries.

The comparison of the GDT-TS of the best structures created by Rosetta shows no statistical difference between PSI and Robetta (*p* = 0.37). This means PSI libraries, generated by Profrager, are a suitable replacement for Robetta as they allowed Rosetta to model the native structure with similar accuracy. Moreover, the rate of correct secondary structures (given by STRIDE [35]) is indistinguishable in practice for these libraries. (Fig 11).

Although BLO fragments were shown to potentially lead to better results (Fig 6), the comparison of the GDT-TS of Robetta and BLO (ANOVA, *p* = 0.0007) shows a significant difference. These results confirm that, in a real *ab initio* scenario, fragment selection is better when biased by secondary structure prediction due to the limitations of the search algorithm. This suggests that the full potential of BLO libraries could not be fully explored by the protocol used by Rosetta and corroborate the view that secondary structure prediction is of special importance in current prediction methods.

Despite the inferior results of the BLO fragments, our analysis of the accuracy of secondary structures demonstrate that the advantages brought by ranking fragments with the aid of PSIPRED are limited to structured regions (Fig 11). Both PSI and Robetta use secondary structure prediction to assemble their fragment libraries, and both had better results for helices and extended conformations (strands) when compared to BLO. Contrastingly, BLO libraries are more accurate in coil regions. Better results in structured regions are obtained when the fragment selection relies on secondary structure prediction (e.g.: PSI, Robetta), but such libraries are prone to exhibit a noticeably smaller structural diversity, to a point where some positions have only one type of secondary structure (Fig 9). Such limited fragment diversity apparently interfered with their ability to model coils correctly, making BLO the proper library for the task of modeling regions that are rich in loops, such as some binding sites.

A useful fragment library should contain all possible conformational states for a local segment of the target protein, albeit without making the search space unfeasible. Local conformational diversity is a desirable trait, as it increases the chance of sampling the correct structure, but too many fragments may cause the search space to become too large for the search algorithm. In short, every position should have as few fragments as possible, but not fewer. Thence, the ideal fragment library combines both BLO and PSI fragments but uses PSI fragments to rebuild regions rich in helices and sheets, while leaving loops to be modeled by BLO fragments.

## Methods

One of the most well-known servers for fragment libraries creation is the Robetta server [36, 37] (http://robetta.bakerlab.org/fragmentsubmit.jsp), which has proven useful for protein structure prediction and protein structure design. However, Robetta does not allow for the customization level we seek to assess in this work, such as varying scoring functions, different fragment sizes and fragment selection method. Therefore, we developed a highly customizable method for fragment library generation to be used by prediction methods. Different fragment libraries can be generated for a target sequence depending on the customization options, which include (i) fragment selection criterion, (ii) fragment length and (iii) number of fragments per library.

### Fragment library creation

The PISCES (Protein Sequence Culling Server [38]) was initially used to extract a non-redundant, and non-homologous, subset of 4365 structures (X-Ray or NMR-determined, 2.0 Å or better resolution) from the RSCB PDB data bank. We used a strict criterion of at most 20% sequence similarity to construct the database. Thus, structural diversity is guaranteed by avoiding the selection of several fragments from homologous proteins.

Fragment libraries were created using the software Profragger (www.lncc.br/sinapad/Profrager/). Each residue is represented by three dihedral angles (*ϕ*, *ψ* and *ω*) and three bond angles [23] (N-C*α*-C, C*α*-C-N, and C-N-C*α*) extracted from the non-redundant database. Fragments were excised exclusively from structures non-homologous to the target sequence (PSIBLAST E-value > 0.05).

Fragment libraries are expected to comprise fragments from non-homologous structures that correctly model a sequential, local segment. Profrager searches the best local structures by aligning overlapping fragment sequences with the query sequence and estimates the likelihood of the fragment via an amino acid substitution matrix. Although Profrager allows the user free choice of the substitution matrix, in this work, we used the BLOSUM62 matrix as it is the *de facto* matrix for protein alignment.

Profrager creates a list starting at every residue of the target sequence, resulting in *n-f+1* lists for a target sequence, where *n* is the number of residues and *f* is the fragment length. Each list contains 200 fragments that better fit the local structure according to the scoring function. For the sake of comparison, two different scoring functions were used to select the fragments. The first function estimates the likelihood of local fit according to the sequence similarity between the target and the fragment sequences. This similarity is given by the BLOSUM62 matrix entries for each pair of residues.

$$\begin{array}{c} \sum_{i=1}^{n}B\left(t_{i},f_{i}\right) \end{array}$$ (1)

where *B* is the value of BLOSUM62 for the residue pair, *t_i_* is the *i*th residue of the target sequence, *f_i_* is the *i*th residue of the fragment, and *n* is the number of residues of the fragment.

The second function tested combines the sequence similarity score with the agreement between the fragment structure prediction for the target sequence, using the PSIPRED prediction confidence (Eq 2).

$$\begin{array}{c} \sum_{i=1}^{n}P\left(t_{i},f_{i}\right),\;\;\;P\left(t_{i},f_{i}\right):\left\{\begin{array}{c} c_{i},\;\text{if}\;ssf_{i}\left(H,E,C\right)=sst_{i}\left(H,E,C\right)\\ -c_{i},\;\text{else} \end{array}\right. \end{array}$$ (2)

where *ssf_i_(H,E,C)* is the secondary structure observed experimentally for the *i*th residue of the fragment (given by STRIDE), *sst_i_(H,E,C)* is the secondary structure predicted to the *i*th residue of the target sequence, *c_i_* is the PSIPRED confidence, *t_i_* is the *i*th residue of the target sequence.

The final score is calculated according to Eq (3)

$$\begin{array}{c} \sum_{i=1}^{n}\left[B\left(t_{i},f_{i}\right)\;+\;P\left(t_{i},f_{i}\right)\right] \end{array}$$ (3)

The weights of B() and P() were determined via trial runs, where some proteins were selected and 10 runs of the algorithm were performed. The best RMSDs were obtained when the weights were 1:1 (S2 Table).

Robetta creates two different sets of fragments for each overlapping position of 3 and 9 residues long, respectively. As Profrager creates fragments of any size, we introduced a fragment of intermediate size (6-mers) because the distance between 3 and 9 is too broad.

For each fragment length, two different ranks were used: the first uses only the sequence similarity score given by Eq (1) and the second uses the score given by Eq (3). As it is a common practice by many PSP algorithms to combine fragments of different lengths, we also investigated the effects of using *mixed libraries*, containing the three fragment lengths.

### Model construction by fragments assembly

A greedy algorithm was used to assemble the native structure solely from overlapping fragments to evaluate the most fundamental aspects of better fragment libraries. A cost-function based on the protein structure was used as it allows fast convergence towards the native structure.

#### Cost function

The Distance Matrix Error (DME, Eq 4) is used as the cost-function.

$$\begin{array}{c} \text{DME}=\sqrt{\sum_{i=1,j>i}^{N}\frac{{\left(p_{ij}-q_{ij}\right)}^{2}}{\frac{N(N-1)}{2}}}, \end{array}$$ (4)

where *N* is the number of backbone atoms (N, C*α* and C) and *p_ij_* (respectively, *q_ij_*) denotes the distance between atoms *i* and *j* on the predicted (respectively, experimental) structure.

Two versions of DME are used in different stages of the algorithm. The *local DME* refers to the DME of a section of the protein, for example, a fragment of 9 residues at some position, while the *global DME* is the DME of the whole structure.

#### Search algorithm

For a *n*-residue long protein, there are 200*^n-f+1^* models that may result from all the possible combinations of the *f*-residue long fragments in a library, making an exhaustive approach unfeasible even for the smallest proteins. We developed a greedy algorithm to assemble the fragments in the close-to-native model, that is composed of three different stages (i) local optimization; (ii) global optimization and (iii) refinement.

The protein starts from a stretched conformation, that is, the backbone dihedral angles are set to 180°. One position on the target sequence is randomly selected and the fragment that minimizes local DME is inserted. The fragment insertion consists in replacing the backbone dihedral and bond angles of the model by those of the fragment. Another position is randomly selected among the positions left, and its fragments are tested for local fit.

start

 until stop criterion

  while j < n_*residues*_

   select random position among n-j

   while i < n_*fragments*_

    evaluate fragment_*i*_

    if DME_*local*, *current*_ < DME_*local*, *best*_

     insert fragment

    end if

   end while

  end while

end

For the global optimization, the algorithm was modified to allow the structure to escape local minima and sample other possible conformations. This is done by testing fragments for two different positions at the same time, and inserting both if the global DME is improved. The global optimization can be summarized as follows: two positions are randomly selected and one fragment is randomly chosen for each position. For each two selected positions, 200 pairs are tested for global DME and the pair that minimizes the global DME is inserted. These positions are marked so they are not sampled again, and two new positions are randomly selected. After the entire sequence has been sampled, the global optimization restarts using the model built as a starting point. The stop criterion is fulfilled when DME is not updated after an iteration, i.e., if the algorithm runs through the entire target sequence and does not insert any pair.

start

 until stop criterion

  while j < n_*residues*_

   select two random positions (a, b) among n-j

   while i < 200

    randomly select fragment_*a*_ and fragment_*b*_

    evaluate fragments

    if DME_*local*, *current*_ < DME_*local*, *best*_

     insert fragments

    end if

   end while

  end while

end

Even though the protein is already folded in a close-to-native conformation after the global optimization, the model can usually be further refined. We introduced a *refinement* stage that is similar to the local optimization, but the cost-function used is the global DME instead of local DME. The algorithm starts with the conformation left by the global optimization and randomly selects a position of the target sequence. All the 200 fragments are tested for global fit, and the best is inserted. The selected position is marked not to be repeated and a new position is randomly selected. After all positions have been selected, the algorithm restarts with the conformation left by the previous iteration and repeats the procedure. The stop criterion is fulfilled when global DME is not updated after an iteration.

When mixed libraries are used, few modifications are introduced on the standard algorithm. A fragment length is randomly selected at the beginning of each stage. The optimization is then performed using only the fragments of the selected length. A new fragment length is then randomly chosen among the options left and the optimization is performed again. For each optimization stage, each fragment library is used only once, in random order.

For each library, 30 runs of the optimization algorithm were performed.

### Fragment clustering

To reduce the search space, we used an algorithm to cluster fragments and verified the effect of rebuilding the native structure solely from the fragments with the highest score in each cluster (called *cluster leader*).

A DME cutoff distance is defined and each fragment is compared to the leader in descending score order. If a fragment does not meet the cutoff, it becomes the leader of a new group; if a fragment meets the cutoff with any existing leaders, it leaves the library and is represented by that leader. This procedure is carried out for each target sequence position. We adopted a DME cutoff of 0.05, 0.7 and 1.5 Å for the 3-mers, 6-mers and 9-mers, respectively.

#### Test set

Tests were carried out in a set of 20 proteins divided among mainly-*α*, mainly-*β* and *α*+*β* classes, ranging from 20 to 129 residues (Table 3).

**Table 3:** Test set.

PDB	Class	Length	PDB	Class	Length
1L2Y	*α*	20	1ENH	*α*	54
1E0N	*β*	27	1FYJ	*α*	57
1AMB	*α*	28	2PLP	*α* + *β*	60
1FSD	*α*	28	1BDD	*α*	60
1PSV	*α*	28	1KOY	*α*	62
1VII	*α*	36	2NR2	*α* + *β*	76
1E0L	*β*	37	1FNA	*β*	91
1I6C	*β*	39	1H5P	*α* + *β*	95
1F4I	*α*	45	2K2B	*α*	111
1BAL	*α*	51	2YVB	*α* + *β*	129

PDB = PDB code; Class = SCOP classification; Length = number of residues

### Profrager fragments with Rosetta

To show that Profrager is an adequate replacement for the fragments generated by the Robetta server, Profrager fragments were tested with the Rosetta suite in an *ab initio* simulation and compared to models created using Robetta fragments. It is only possible to test Profrager 9-mers and 3-mers as these are the only fragment sizes used by Robetta.

A set of 48 proteins ranging from 54 to 148 residues was selected from the CASP9 experiment (Table 2) and for each we generated 1000 models using the *ab initio*-relax protocol (Rosetta v3.4). The quality of the structures was evaluated by GDT-TS (Global Distance Test Total Score [39, 40], given by TM-score [41, 42]) and only the best model (greater GDT-TS) was considered during comparisons.

### Statistical test

In each test, the best models for each protein were compared. One-way analysis of variance (ANOVA) was performed with the aid of Scipy package (v.0.18.1-1) for the Python language (v.3.5.2). The corresponding p-values for each test are cited on the Results section. Differences were considered significant when *p* < 0.01.

## Conclusion

Despite the fact that fragment libraries are commonly used in protein structure prediction, some of its underlying features have never been fully investigated. To have a clearer view of how distinct factors associated to fragment generation and use affect protein structure prediction, we isolated the process of model building by fragment assembly from some common limitations associated with prediction methods, e.g., imprecise energy functions and optimization algorithms, by employing an exact structure-based objective function under a greedy algorithm.

Firstly, we assessed the effect of varying fragment sizes in libraries and found that, when considered separately, shorter fragments produce better models than longer fragments. Notwithstanding, combining fragments of all sizes into a mixed library proved to be the best strategy. When mixed libraries are used, longer fragments are invaluable at the beginning of the simulation because they bring more empirical information into the model. These findings suggest the best approach is to use a library with different fragment sizes and to diminish their size as the simulation advances to fully exploit them.

A library composed of 3,6,9-mers has the potential to reproduce the native structure with the same degree of accuracy of a library with fragments of sizes 1 to 20 residues. There is a high degree of structural redundancy among fragments of similar sizes, which makes it possible to remove them from the library and, by consequence, to explore the search space more thoroughly.

Secondly, the fragment libraries were evaluated regarding the fragment selection criteria. Fragments could be selected exclusively by sequence similarity given by a BLOSUM62 score (BLO libraries) or by combining the BLOSUM62 score to the secondary structure prediction confidence provided by PSIPRED (PSI libraries). The former approach yielded closest to native models. Despite the common reliance on PSIPRED predictions, our results strongly indicate that its use in fragment ranking can exclude useful fragments, thus casting reservations on the widespread utilization of secondary structure prediction for fragment libraries construction.

Libraries assembled with the aid of PSIPRED were found to be less diverse, and while they may exclude some useful fragments, they allow better structural clustering and a much greater reduction of the search space. The relatively smaller number of clusters of PSI libraries shows that using secondary structure prediction for fragment selection is more suitable to an optimization problem where the conformational sampling is the main limiting aspect of the model construction process, as is the case of *ab initio* prediction.

Finally, though lacking conformational diversity PSI libraries can lead to better results in structured regions (helices and sheets). Altogether, these results suggest that fragment libraries should couple PSI and BLO fragments to model structured regions and loops, respectively.

Profrager PSI libraries proved to be a suitable replacement for Robetta, as it generates fragment libraries that allow Rosetta to recreate the native structure with similar accuracy.

The results shown will guide the development of strategies for generation and use of fragment libraries in our protein structure prediction methods [43]. Furthermore, these results prompt the development of novel strategies to build fragment libraries by mixing the best features of sequence similarity based and secondary structure based libraries. Such strategy could be the construction of a library that increases the weight of the sequence similarity score in regions where the probability of coils is detected to be high. Another possible approach could be the use of a multi-objective strategy to select the fragments, such as a Pareto strategy.

## Supporting Information

S1 Table **BLO**: fragment libraries generated using only sequence similarity calculated with BLOSUM62. **PSI**: libraries generated using sequence similarity calculated with BLOSUM62 and PSIPRED secondary structure prediction of the target sequence.
(PDF)

S2 Table **Trial runs**: best RMSDs (min) and average RMSDs (avg) for 10 runs with different weights for the PSIPRED score (*P*) relative to the BLOSUM62 score (*B = 1*).
(PDF)

S1 Fig Relative frequency of fragment insertion. The bars show the relative amount of use of each fragment size (from 3 to 20-mers) in different stages of the algorithm when mixed libraries are used. Proteins are shown as numbers and are sorted according with size (Table 3).
(TIF)
