# Supplementary material for: Critical Features of Fragment Libraries for Protein Structure Prediction
Source: PLoS One. 2017 Jan 13;12(1):e0170131. doi: 10.1371/journal.pone.0170131 (PMC5235372; doi:10.1371/journal.pone.0170131)
Supplement: S1 Table — BLO: fragment libraries generated using only sequence similarity calculated with BLOSUM62. PSI: libraries generated using sequence similarity calculated with BLOSUM62 and PSIPRED secondary structure prediction of the target sequence. (PDF) [file pone.0170131.s001.pdf]

S1 Table

		RMSD(Å)							
		**3-res**		**6-res**		**9-res**		**9,6,3-res**	
**PDB**	**Length**	**BLO**	**PSI**	**BLO**	**PSI**	**BLO**	**PSI**	**BLO**	**PSI**
1L2Y	20	1.00	2.10	2.49	2.83	5.79	5.78	0.79	1.86
1E0N	27	1.64	2.20	2.93	2.78	4.92	4.80	1.37	1.65
1AMB	28	1.72	2.35	2.64	2.29	2.77	2.85	2.12	2.11
1FSD	28	1.26	1.46	3.98	4.12	5.56	5.66	1.20	1.34
1PSV	28	1.53	1.53	3.88	4.58	5.58	5.29	1.46	1.45
1VII	36	2.18	1.14	3.23	2.73	4.26	4.30	1.06	1.36
1E0L	37	1.97	2.31	2.71	2.70	4.23	4.04	1.82	2.00
1I6C	39	2.17	2.05	4.02	3.99	5.36	4.06	1.81	2.18
2P81	44	1.73	3.07	2.46	3.12	3.55	4.68	1.60	2.42
1BAL	51	2.93	2.27	3.33	3.50	4.77	4.82	2.27	2.68
1ENH	54	2.71	1.86	2.38	2.39	3.88	3.62	1.31	1.39
2PLP	54	2.88	3.31	2.58	3.68	5.51	5.89	1.32	2.84
1FYJ	57	2.50	1.48	1.80	1.82	2.93	2.67	1.09	1.09
1BDD	60	3.26	2.07	2.66	2.92	3.98	4.10	1.38	1.65
1KOY	62	2.79	1.82	2.81	2.66	4.40	4.20	1.53	1.40
2NR2	76	3.59	3.54	3.12	3.65	3.14	3.65	1.66	2.01
1FNA	91	4.19	3.77	3.74	3.93	4.25	3.34	3.77	2.42
1H5P	95	4.68	4.09	4.74	4.48	4.91	5.65	3.39	3.67
2K2B	111	4.52	5.02	4.66	5.64	4.94	5.31	3.67	3.99
2YVB	129	5.71	5.93	3.74	4.76	3.91	5.60	2.18	3.81
