# Supplementary material for: Critical Features of Fragment Libraries for Protein Structure Prediction
Source: PLoS One. 2017 Jan 13;12(1):e0170131. doi: 10.1371/journal.pone.0170131 (PMC5235372; doi:10.1371/journal.pone.0170131)
Supplement: S2 Table — Trial runs: best RMSDs (min) and average RMSDs (avg) for 10 runs with different weights for the PSIPRED score (P) relative to the BLOSUM62 score (B = 1). (PDF) [file pone.0170131.s002.pdf]

		RMSD(Å)			
PDB	P	0.25	0.50	0.75	1.00
1E0L	min	3.63	3.53	3.90	3.52
	avg	4.77	4.44	4.49	4.99
1FYJ	min	3.21	3.09	3.12	3.01
	avg	4.61	4.71	4.46	4.32
1I6C	min	3.86	4.52	3.74	3.49
	avg	5.30	6.02	5.73	4.48
1BDD	min	3.71	3.47	3.42	3.15
	avg	5.66	4.25	4.88	4.84
