# Supplementary figures and images for: Critical Features of Fragment Libraries for Protein Structure Prediction
Source: PLoS One. 2017 Jan 13;12(1):e0170131. doi: 10.1371/journal.pone.0170131 (PMC5235372; doi:10.1371/journal.pone.0170131)

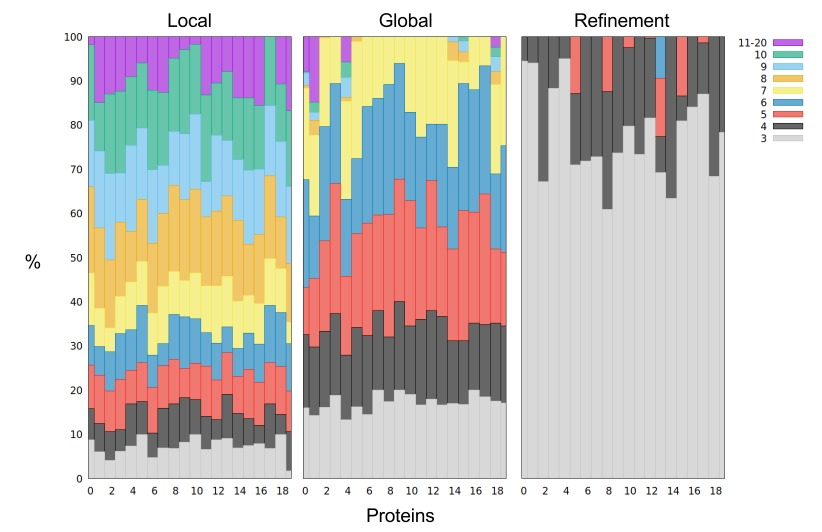

Supplement: S1 Fig — The bars show the relative amount of use of each fragment size (from 3 to 20-mers) in different stages of the algorithm when mixed libraries are used. Proteins are shown as numbers and are sorted according with size (Table 3). (TIF) [file pone.0170131.s003.tif]
